# Identification and Characterization of Plasmids and Genes from Carbapenemase-Producing *Klebsiella pneumoniae* in Makkah Province, Saudi Arabia

**DOI:** 10.3390/antibiotics11111627

**Published:** 2022-11-15

**Authors:** Rayan Y. Booq, Mohammed H. Abutarboush, Mohammed A. Alolayan, Abdulaziz A. Huraysi, Amjad N. Alotaibi, Maha I. Alturki, Maryam K. Alshammari, Abrar A. Bakr, Azzam A. Alquait, Essam A. Tawfik, Nasser B. Alsaleh, Fayez S. Bahwerth, Mohammed S. Alarawi, Essam J. Alyamani, Bandar K. Sendy

**Affiliations:** 1 National Centre for Biotechnology, Life Science and Environment Research Institute, King Abdulaziz City for Science and Technology (KACST), Riyadh 12354, Saudi Arabia; 2 Department of Botany and Microbiology, College of Science, King Saud University, Riyadh 12371, Saudi Arabia; 3 Department of Pharmacology and Toxicology, College of Pharmacy, King Saud University, Riyadh 12372, Saudi Arabia; 4 Microbiology Department, King Faisal Hospital, Ministry of Health, Makkah 24236, Saudi Arabia; 5 Computational Bioscience Research Center, King Abdullah University of Science and Technology (KAUST), Thuwal 23955, Saudi Arabia

**Keywords:** bacterial infection, hospital-acquired infections, multidrug-resistant, *Klebsiella pneumoniae*, carbapenemases, plasmid

## Abstract

*Klebsiella pneumoniae* (*K. pneumoniae*) is involved in several hospital and community-acquired infections. The prevalence of *K. pneumoniae*-producing-carbapenemase (KPC) resistance genes rapidly increases and threatens public health worldwide. This study aimed to assess the antibiotic resistance level of *K. pneumoniae* isolates from Makkah Province, Saudi Arabia, during the Islamic ‘Umrah’ ritual and to identify the plasmid types, presence of genes associated with carbapenem hydrolyzing enzymes, and virulence factors. The phenotypic and genotypic analyses based on the minimum inhibitory concentration (MIC), biofilm formation, PCR, and characterization of KPC-encoding plasmids based on the replicon typing technique (PBRT) were explored. The results showed that most isolates were resistant to carbapenem antibiotics and other antibiotics classes. This study identified sixteen different replicons of plasmids in the isolates and multiple genes encoding carbapenem factors, with *bla_VIM_* and *bla_OXA-48_* being the most prevalent genes identified in the isolates. However, none of the isolates exhibited positivity for the KPC production activity. In addition, this study also identified six virulence-related genes, including *kfu*, *wabG*, *uge*, *rmpA*, *fimH,* and a capsular polysaccharide *(CPS)*. Together, the data reported in this study indicate that the isolated *K. pneumoniae* during the pilgrimage in Makkah were all resistant to carbapenem antibiotics. Although the isolates lacked KPC production activity, they carried multiple carbapenem-resistant genes and virulence factors, which could drive their resistant phenotype. The need for specialized methods for KPC detection, monitoring the possibility of nosocomial transmission, and diverse therapeutic alternatives are necessary for controlling the spreading of KPC. This study can serve as a reference for clinicians and researchers on types of *K. pneumoniae* commonly found during religious gathering seasons in Saudi Arabia.

## 1. Introduction

*Klebsiella pneumoniae (K. pneumoniae) is an encapsulated, non-motile gram-negative nosocomial pathogenic* [1,2]. This bacterium involves several disseminated hospital and community-acquired infections, such as pneumonia, septicemia, surgical site infections, and urinary tract infections (UTIs) [3]. *K. pneumoniae* belongs to the ESKAPE pathogenic group, which includes *Enterococcus faecium*, *Staphylococcus aureus*, *K. pneumoniae*, *Acinetobacter baumannii*, *Pseudomonas aeruginosa*, and *Enterobacter* species, which escapes the antimicrobial drugs through multi-action mechanisms [4,5]. The evolution of multidrug-resistant infections (MDR) has been facilitated by the improper use of antimicrobial medications over time, which led to limited treatment options [6]. Given this information, previous research indicated that the mortality rate from severe infections exceeded 40%. This high prevalence is associated with carbapenem-resistant *K. pneumoniae* (CRKP) strains, which are primarily hospital-acquired infections (HAIs) [7,8,9]. In 2017, the World Health Organization (WHO) classified and listed CRKP among the global pathogens that pose an unprecedented challenge in developing new antibiotics [10].

*K. pneumoniae*-producing-carbapenemase (KPC) was first identified in the first decade of the 21st century [11]. Since then, the KPC-encoding gene has been noticed frequently in several regions, including Europe, Asia, and the Middle East [12]. The emergence of K. pneumoniae harboring carbapenemase resistance genes posed a significant threat to global public health [13]. In recent years, extensive dissemination, rapid spreading, and failure to treat KPC infections have been associated with multiple resistance mechanisms. For instance, alteration of the bacterial target site, inactivation of the antimicrobial metabolic pathways, reduction of antibiotic accumulation through efflux pump systems, and biofilm formation [14]. It should be noted that plasmid-mediated carbapenemase genes are one of the most concerning mechanisms of antibiotic resistance by *K. pneumoniae* [15]. Its ability to anchor and rapidly spread MDR elements allows it to attain numerous β-lactamase enzymes that can break down the β-lactam ring in strong antibiotics, such as carbapenems used as the final line of infection therapy [16].

Based on the Ambler classification system, carbapenemases are divided into A, B, and D β-lactamase classes [17]. Class C possesses chromosome-encoded cephalosporinase, producing insignificant effects against carbapenems [17]. Class A carbapenemases are encoded by chromosomes, such as Sme, SFC-1, IMI-1, and NmcA. The plasmid encodes other classes, namely KPCs, GES, and IMI-2 [18]. These carbapenemases are effective against carbapenems, but the most clinically prevalent is the KPC [19]. From the molecular level of class B β-lactamases, these enzymes have active-site zinc, making them metalloenzymes [20]. The most common families of metallo-β-lactamases are GIM, IMP, SIM, and VIM. Another metallo-β-lactamase, NDM-1, was discovered in 2009 in clinical isolates of *K. pneumoniae* and *Escherichia coli* in India [21]. This new family of carbapenems enzymes poses a high threat to community health due to the rapid dissemination of the *bla*_NDM_ gene in isolates [22]. Class D β-lactamases contain more than 350 OXA-enzymes, also known as oxacillinases (OXA-type enzymes) [23]. This class of β-lactamases includes carbapenem-hydrolyzing class D β-lactamases (CHDLs) or Class D β-lactamases that are resistant carbapenem antibiotics. Several subgroups of CHDLs are identified according to their amino acid sequence identity. The most disseminated enzyme subgroups of CHDLs in bacterial pathogens are OXA-23, OXA-24/40, OXA-48, OXA-51, OXA-58, and OXA-143 [14]. OXA-48 carbapenemase is the enzyme with the highest virulence factors among other subgroups and is presented in *K. pneumoniae* and other *Enterobacteriaceae* family members. The rest of the enzyme subgroups can mainly be found in *Acinetobacter* isolates [24].

Virulence genes play a vital role in *K. pneumoniae*’s pathogenicity. These genes encode virulence factors such as hypermucoviscosity, lipopolysaccharide formation, capsule synthesis, adhesion, and iron uptake systems [25,26]. A positive string test identifies hypervirulent *K. pneumoniae* (hvKp) isolates, which harbor hypermucoviscosity colonies [27]. This phenotype was confirmed by two genes, *rmpA* and *magA*, that regulate capsule polysaccharide synthesis and mucoviscosity [28]. Among the 79 defined capsular serotypes in previous studies, capsule serotypes K_1_ and K_2_ are the most common and frequently associated with the *rmpA* gene, allowing bacteria to evade the immune system [29]. Another gene that regulates capsule formation, hypermucoviscosity, and iron transport system is the *Klebsiella ferric iron uptake* (*Kfu*) [30]. *wabG* and *uge* are two other hypervirulence genes that promote lipopolysaccharides synthesis and robust *K. pneumoniae* resistance [31]. Moreover, the *fimH* gene regulates fimbriae protein and the bacteria cell’s adhesion [32]. Consequently, the most concerning aspect of *K. pneumoniae* is the dual-risk isolates which produce carbapenemases and carry hypervirulence genes [33].

This study describes the characteristics of the antibiotic-resistant *K. pneumoniae* isolates from Makkah Province, Saudi Arabia, during the Islamic pilgrimage ‘Umrah’ ritual. It aims to identify the plasmid types, the presence of genes associated with carbapenem hydrolyzing enzymes and virulence factors, and to determine antibiotic susceptibility by performing the minimum inhibitory concentration (MIC) assay. The study may serve as a potential reference for clinicians and research scientists to understand the nature of *K. pneumoniae* KPC, that are common in religious gathering seasons in Saudi Arabia. This study also highlights the need for rapid methods for KPC detection, monitoring the possibility of nosocomial transmission, and exploring novel therapeutic alternatives for controlling the spreading of KPC.

## 2. Results and Discussion

### 2.1. Bacterial Identification and Antibiotic Susceptibility Test

A total of 23 carbapenems-resistant *K. pneumoniae* (CRKP) strains were collected from different patients in King Faisal Hospital in Makkah province, Saudi Arabia, during the religious gathering season of Umrah. Samples were isolated from sputum, wounds, urine, blood, and central venous puncture, with ratios of 43%, 22%, 17%, 13%, and 4%, respectively. The isolates’ identification confirmation was processed using a MicroScan and VITEK2. The MIC of the nine antibiotics used against the isolates and the ATCC reference strain (BAA 1705) showed a remarkable resistance to carbapenems drugs. Most isolates exhibited resistance against FOX, CPM, AZT, CAZ, CTC, CIP, AMP, and most importantly against MEM and IMI, all demonstrating MIC of >1024 µg/mL, as shown in Table 1. However, there was only one isolate (K5) that showed an intermediate susceptibility for FOX (MIC = 16 µg/mL) and CIP (MIC = 2 µg/mL), while the MIC was <0.5 µg/mL against AZT, CAZ, and CPM. The same isolate had above-the-threshold resistance levels for CTC, IMI, and AMP (MIC ≥ 1024 µg/mL). Two strains, K5 and K21, are susceptible to MEM (MIC < 0.5 µg/mL) among the 23 isolates. Additionally, isolate K9 exhibited a sensitivity for IMI at MIC = 2 µg/mL, as shown in Table 1. The study showed that MEM was considered more effective than IMI, which 33% of samples resistant to IMI (MIC of >1024 µg/mL). These results are consistent with a study by Subash et al., who reported that the sensitivity of Gram-negative bacilli to MEM is more than IMI due to the overuse of the latter antibiotic (for many years) as an empirical treatment option [34].

### 2.2. Biofilm Formation of K. pneumoniae

All 23 *K. pneumoniae* isolates and the ATCC reference strain (700603) were tested for their ability to form biofilms, as shown in Table 2. The biofilm formation of the 23 isolates was interpreted by four standard criteria for producing biofilm, as shown in Table 2. The findings showed that nine isolates (≈39.13%) were non-biofilm-forming strains, while eleven were weak biofilm-forming strains (≈8.83%). In addition, only three isolates out of the 23 (≈13.04%) formed biofilm moderately. There was no evidence of strong biofilm formation for all 23 isolates (0%) (Table 2). The ATCC strain (700603) showed moderate biofilm formation ability. More details on the biofilm formation level of all *K. pneumoniae* strains are shown in the Supplementary Materials Section (Table S1).

This table shows that 13% of all collected isolates could moderately form a biofilm, which they initially collected from the sputum and wound. By comparison, other isolates (≈87%) were collected from the sputum, blood, wound, urine, and central venous puncture. There was no relationship between the source of isolates and the biofilms-forming. Ashwath et al. reported that the ability of bacteria to form biofilms is not necessarily involved in the source of isolation [36]. It is important to note that 39% of the non-biofilm-forming isolates have high resistance to carbapenem antibiotics, which could indicate a lack of association between biofilm formation and resistance to carbapenem antibiotics. This latter might occur due to other antibiotic-resistance factors. Further studies are needed to confirm the irrelevance between biofilm formation and carbapenem antibiotics resistance.

### 2.3. Detection of Plasmids by PCR-Based Replicon Typing (PBRT)

**Figure 1:**
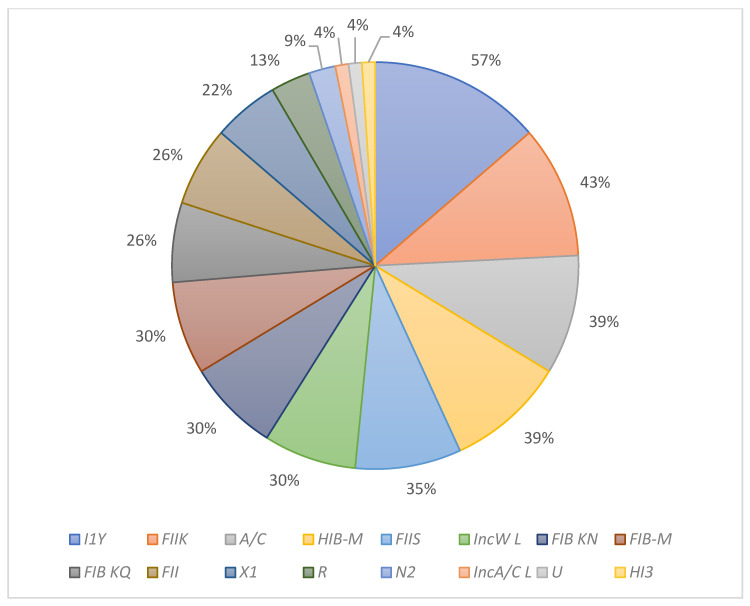
The percentage of replicons in *K. pneumoniae* KPC isolates.

Thirty replicons were amplified using eight multiplex PCR assays, as shown in Table 3. for plasmid identification in *K. pneumonia* isolates owing to its efficiency and low cost compared to traditional methods, such as conjugation or molecular cloning [37]. PBRT uses a set of primers and a probe specific to the target plasmid to generate amplicons of different molecular weights. The different amplicon sizes correspond to different plasmids and can be used to identify a particular plasmid. Here, PBRT was used to determine the plasmids in KPC *K. pneumoniae* isolates. Sixteen replicons (with their percentage of appearance) out of thirty were presented in this study as follows: I1Y (57%), FIIK (43%), A/C (39%), HIB-M (39%), FIIS (35%), IncW L (30%), FIB KN (30%), FIB-M (30%), FIB KQ (26%), FII (26%), X1 (22%), R (13%), N2 (9%), HI3 (4%), IncA/C L (4%), and U (4%) (Figure 1). More detailed results are shown in the Supplementary Materials Section—Table S2. A previous study showed seven types of replicons in the *K. pneumoniae* isolates [38].

Plasmids are considered one of the significant factors of antibiotic resistance, including the carbapenem group [39,40]. The study samples showed that they contain a high number of plasmids. A recent study on *K. pneumoniae* pathogenic strains’ genetic characteristics demonstrated that such bacterium usually has many plasmids that can cause diseases [39]. A previous study conducted in Riyadh, Saudi Arabia, between 2011 and 2012 observed that *K. pneumoniae* isolates contained four plasmids, the most popular was FIIK, reaching 69% [38]. However, in our study, this plasmid appeared in 43% of the tested isolates. Additionally, the study identified a group of isolates containing more than seven plasmids, which reached ten plasmids in one isolate. It is worth noting that four isolates do not contain plasmids (K5, K7, K11, and K13) and have shown high resistances to both MEM and IMI, which may be due to the limited plasmids that can be monitored through the PBRT mechanism used in this study, which needs further investigation.

### 2.4. Genotyping Characterization for Harboring Carbapenems Genes

The presence of gene-encoding carbapenems factors (*bla_KPC_*, *bla_OXA-48_*, *bla_VIM_, bla_NDM-1_, bla_IMP_-*variants, and *bla_OXA-23-_*like) was investigated by PCR. Gene *bla_VIM_* was the most prevalent among the studied isolates, and it was presented in all tested isolates (100%), followed by *bla_OXA-48_* gene in 87% of the isolates, *bla_NDM-1_* gene in 30% of the isolates, and *bla_OXA-23_*-like gene in 4% of the isolates (Table 4). Both *bla_KPC_* and *bla_IMP_* genes are missing in all tested isolates (0%).

A reference study conducted in Saudi Arabia on a group of samples collected during the years 2010 to 2018 indicated a low prevalence of the *VIM* gene among the study *K. pneumoniae* isolates; during this study, a percentage of gene presence of 100% for all study samples. On the other hand, no isolates exhibited positivity for the KPC production activity, as shown in Table 5. The KPC enzyme is considered one of the non-dominant genes in the *K. pneumoniae* bacterium isolated in Saudi Arabia, which was previously monitored in studies conducted on wastewater [41,42].

Several studies have shown the possibility of *K. pneumoniae* carrying more than one carbapenem resistance gene in isolates from China, Singapore, India, Europe, and the Middle East [43,44,45]. In this study, all isolates showed the coexistence of at least two carbapenems genes, except for isolates K5 and K21, which produced the *bla_VIM_* gene only. Another study in Egypt showed the appearance of *OXA-48* and *NDM* genes in 13 of the study *K. pneumoniae* isolates [46]. In contrast, this study recorded that five isolates (K2, K6, K8, K10 and K12) carried three carbapenem-resistant genes, *bla_OXA-48_*, *bla_VIM_* and *bla_NDM-1_* (Table 5). A study conducted in the Makkah region in Saudi Arabia reported that *K. pneumoniae* contains only three carbapenem-resistant genes [44]. However, our study indicated that this bacterium could hold more than three resistant genes (isolate K22), which held four carbapenem resistance genes blaOXA-48, blaVIM, blaNDM-1, and blaOXA-23. More investigations are required to connect the number and type of genes against antibiotic resistance.

### 2.5. Detection of Virulence Factors

**Figure 2:**
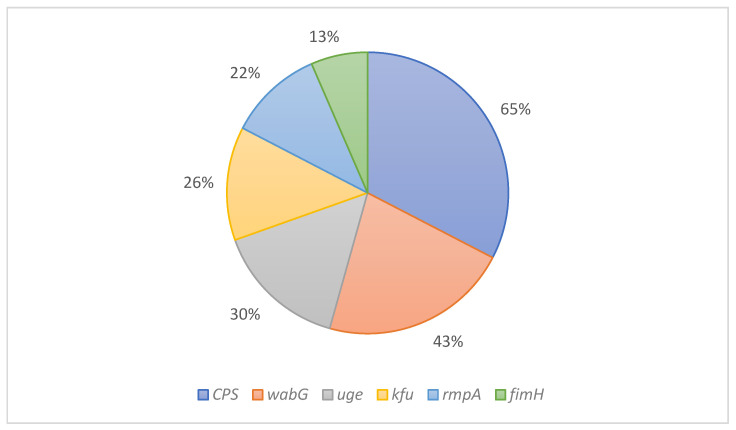
The percentage of virulence factors in *K. pneumoniae* KPC isolates.

Virulence factors indicate chromosomally encoded genes involved in the bacterium’s attachment to host tissues and the ability to produce toxins that cause diseases. This study identified six virulence-related genes (*kfu*, *wabG*, *uge*, *rmpA*, *fimH,* and *CPS*), with a percentage of gene presence of 65%, 43%, 30%, 26%, 22%, and 13%, respectively (Figure 2).

The capsular polysaccharide gene (i.e., *CPS*) is a shared gene within *K. pneumoniae* isolates [29]. One study showed that this gene was present in all isolates (100%) of the study (*n* = 65 isolates) [47], while it appeared in 65% of our study isolates. *wabG* gene in *K. pneumoniae* is a virulence factor involved in the attachment of bacterium to host tissues [48], and it is present in 43% of the isolates in this study. Another study conducted on 23 samples in Saudi Arabia isolated from 2011 to 2015 showed that the prevalence of the *Kfu* gene was 35% [49]. However, our study exhibited a decrease in the number of isolates carrying the *kfu* gene to 26%. In recent years, few studies in Saudi Arabia shed light on *the rmpA* gene, which demonstrated a higher presence rate (35%) than what was shown in this study (22%). Finally, the *fimH* gene was presented in 13% of the isolates in our study, which appeared in 22% in a previous study conducted in the Western region of Saudi Arabia [50]. All six genes appeared in all tested isolates but varied ratios. Further research is required to identify more virulence factor genes using whole genome sequencing on a more significant number of isolates than in this study (*n* = 23 isolates).

## 3. Materials and Methods

### 3.1. Materials

Mueller-Hinton broth (MHB) was purchased from Scharlau (Barcelona, Spain), while LB agar was bought from Invitrogen (USA). Nine antibiotics, including cefoxitin (FOX), cefepime (CPM), aztreonam (AZT), ceftazidime (CAZ), cefotaxime (CTC), ciprofloxacin (CIP), meropenem (MEM), imipenem (IMI), and ampicillin (AMP) were obtained from Sigma-Aldrich (Gillingham, UK), except for AMP which was bought from USB (Cleveland, OH, USA). Acetic acid and ethanol were purchased from BDH (Prolabo, UK) and Scharlau (Barcelona, Spain), respectively. TAE 10X buffer was obtained from Thermo Fisher Scientific (Waltham, MA, USA). Distilled water was generated using Milli-Q^®^ IQ 7005 Purification System (Millipore SAS, Molsheim, France) and was used throughout this study.

### 3.2. Bacterial Inoculum Preparation

Twenty-three carbapenems-resistant *K. pneumoniae* (CRKP) strains were isolated from blood, sputum, wounds, central venous puncture, and urine culture from King Faisal Hospital in Makkah, Saudi Arabia (Table 3). An ATCC BAA 1705 strain (with KPC gene) and ATCC 700603 were used as positive controls. The isolates’ identification and confirmation were assessed using MicroScan (Beckman Coulter, CA, USA) and VITEK 2 (bioMérieux, Marcy-l′Étoile, France) according to the manufacturer’s instructions.

### 3.3. Minimum Inhibitory Concentration (MIC) Assay

The MICs of nine antibiotics that include stock solutions of FOX (5000 μg/mL), CPM (5000 μg/mL), AZT (5000 μg/mL), CAZ (5000 μg/mL), CTC (5000 μg/mL), CIP (5000 μg/mL), IMI (5000 μg/mL), MEM (10,000 μg/mL), and AMP (10,000 μg/mL) against *K. pneumoniae* were determined using the microdilution method. A serial dilution of the drugs, as a half-fold dilution from 1024 to 0.5 μg/mL, in MHB was added into 96-well microtiter plates at a final volume of 100 μL in each well. Then, single pure colonies from all isolates, to create bacterial inoculums using 0.5 McFarland standard, giving a cell density of 1.5 × 10^8^ colonies forming unit (CFU)/mL, were measured by DensiChek Plus Instrument (bioMérieux, Marcy L’Etoile, France) at 600 nm. The bacterial inoculums were added to each well (100 μL) to attain a final inoculum of 1 × 10^6^ CFU/mL. All 96-well microtiter plates were incubated overnight at 37 °C with a continuous shaking speed of 140 RPM. The endpoints of the MIC were measured at a UV absorbance of 600 nm using a PowerWave XS2 plate reader (bioMérieux, Marcy L’Etoile, France) [51]. All the results were evaluated according to the Clinical and Laboratory Standards Institute (CLSI) criteria for Antimicrobial Susceptibility Testing [35]. Wells contained bacterium, and medium only were used as positive and negative controls, respectively.

### 3.4. Biofilm Formation of K. pneumoniae

Biofilm formation assay was performed by allowing the cells to adhere to the walls and the bottom of the 96-well microtiter plates following a modified method [52,53]. *K. pneumoniae* bacterial strains (23 isolates) were cultured on an LB agar plate and incubated overnight at 37 °C. Then, a 10^6^ CFU/mL dilution in LB broth was inoculated into a 96-well microtiter plate and incubated at 37 °C overnight. After the incubation, the plate was washed three times with distilled water using BioTek ELx50 Microplate Strip Washer (BioTek, Winooski, VT, USA) to remove the bacterial suspension and unattached cells. A 125 μL of 0.1% crystal violet (CV) solution was added to each well. The plate was incubated at room temperature for 10–15 min, followed by three thorough washings with distilled water. Finally, the plate was turned upside-down to dry completely at room temperature for 30 min. To quantify the biofilm formation, a 125 μL of 30% acetic acid was added to each well to dissolve the CV-stained biofilm and incubated at room temperature for 10–15 min. The 125 μL solubilized CV-stained biofilm was transferred to a new 96-well microtiter plate, and the absorbance was measured using a PowerWave XS2 plate reader (BioTek, Winooski, VT, USA) at 550 nm. The biofilm formation was quantified by comparing the optical densities cut-off (ODc) with the negative control following the previous studies [54,55]:

ODc = average OD of negative control + (3 × standard deviations (SD) of negative control OD)(1)

The classification of biofilm formation, along with the optical density (OD), for all bacterial isolates was interpreted according to the criteria of [52,53,54], as shown in Table 6.

### 3.5. Bacterial DNA Extraction

The bacterial genomic DNA extraction was obtained using a modified boiling method [56]. Pure bacterial colonies were collected from all isolates and added to 100 µL of sterilized distilled water. The bacterial inoculums were boiled using Eppendorf Thermomixer (Thermo Fisher Scientific, Waltham, MA, USA) at 99 °C for 5 min. The tubes were placed on ice for 3, then centrifuged at 1300 rpm for 20 min. An 80 µL of the supernatant containing DNA was transferred into new Eppendorf tubes, and 160 µL cold absolute ethanol was added; the mixture was centrifuged at 1300 rpm for 5 min. The supernatant was removed, 100 µL of 70% ethanol was added to wash the pellet, followed by further centrifugation at 1300 rpm for 5 min, and then the supernatant was discarded. The pellet-containing genomic DNA was allowed to dry out completely, then re-suspended in 50 µL of nuclease-free water. The concentrations and DNA purities were evaluated spectrophotometrically using QuickDrop (Molecular Devices, San Jose, CA, USA).

### 3.6. Detection of Plasmids by PCR-Based Replicon Typing (PBRT)

PCR-based replicon typing technique (PBRT) package was used to detect the resistant plasmids of the *K. pneumoniae* isolates using PBRT 2.0 kit (Diatheva, Italy). Whole bacterial DNA was obtained via the boiling method and was used as a PCR template. Thirty Replicons were amplified using eight multiplex PCR assays, including HI-1, HI-2, I-1 alpha, I-2, X-1, X-2, X-3, X-4, L, I1y, N, FI-A, FI-B, FI-C, FII, FII-S, FII-K, FIB KN, FIB KQ, W, Y, P1, A/C, T, K, U, R, B/O, HIBM, and FIBM (Table 7). These replicons demonstrate the main plasmid incompatibility groups and replicase genes identified in the resistance plasmids among *Enterobacteriaceae*. The kit also contained positive controls for all particular replicons. PCR conditions were conducted in a thermal cycler (Thermo Fisher Scientific, Waltham, MA, USA) with some modifications as follows: initial denaturation at 95 °C for 10 min, followed by 30 cycles of denaturation at 95 °C for 60 s, annealing at 60 °C for 30 s, extension step at 72 °C for 60 s, and a final extension step at 72 °C for 5 min. The final PCR products were analyzed using electrophoresis at 80 V for 45 min in a 2.5% agarose gel with SYPR safe (Thermo Fisher Scientific, Waltham, MA, USA). The bands were visualized using a UV transilluminator (Bio-Rad Laboratories, Hercules, CA, USA).

### 3.7. Detection of Carbapenem Genes by Polymerase Chain Reaction (PCR)

Conventional PCR was used to detect the presence of carbapenem resistance genes of CRKP isolates, including *KPC*, *IMP*, *OXA 48*, *OXA 23*, *VIM*, and *NDM-1* (Macrogen, Seoul, Republic of Korea). The primers sequence and amplicon sizes represented in this study are shown in Table 8. Multiplex PCR mixture 1 *(KPC*, *OXA-48*, *VIM)* and Multiplex PCR mixture 2 (IMP, OXA-23, NDM) carried out in 25 µL contained 1X PuRE Taq™ Ready-To-Go™ PCR beads Master Mix (GE Healthcare, Amersham, UK),1 µL of each forward primer (10 pmol), 1 µL of each reverse primer (10 pmol), 1 µL of the DNA template, and 18 µL nuclease-free water. The PCR conditions were conducted in a thermal cycler (Thermo Fisher Scientific, Waltham, MA, USA) with some modifications as follows: multiplex PCR 1, initial denaturation at 95 °C for 5 min, followed by 35 cycles of denaturation at 95 °C for 1 min, annealing at 56 °C, extension step at 72 °C for 1 min, and a final extension step at 72 °C for 5 min. For multiplex PCR 2, initial denaturation at 95 °C for 5 min, followed by 35 cycles of denaturation at 95 °C for 1 min, annealing at 52 °C, extension step at 72 °C for 1 min, and a final extension step at 72 °C for 5 min [57]. The final PCR products were analyzed using electrophoresis at 80 V for 45 min in a 2% agarose gel with SYPR safe (Thermo Fisher Scientific, Waltham, MA, USA). The bands were visualized using a UV transilluminator (Bio-Rad Laboratories, Hercules, CA, USA).

### 3.8. Detection of Virulence Genes by Polymerase Chain Reaction (PCR)

Conventional PCR was used to detect the presence of virulence genes of CRKP isolates, including *kfu*, *wab G*, *uge*, *rmpA*, *fimH*, *magA*, and CPS (Macrogen, Seoul, Republic of Korea). The primers sequence and amplicon sizes represented in this study are shown in Table 9. PCR mixture carried out in 25 µL contained 1X PuRE Taq™ Ready-To-Go™ PCR beads Master Mix (GE Healthcare, Amersham, UK), 1 µL of the forward primer (10 pmol), 1 µL of the reverse primer (10 pmol), 1 µL of the DNA template, and 22 µL nuclease-free water. The PCR conditions were conducted in a thermal cycler (Thermo Fisher Scientific, Waltham, MA, USA) with some modifications: initial denaturation at 95 °C for 5 min, followed by 35 cycles of denaturation at 95 °C for 30 s, annealing at 60 °C, extension step at 72 °C for 5 min, and a final extension step at 72 °C for 7 min [59]. The final PCR products were analyzed using electrophoresis at 80 V for 45 min in a 2% agarose gel with SYPR safe (Thermo Fisher Scientific, Waltham, MA, USA). The bands were visualized using a UV transilluminator (Bio-Rad Laboratories, Hercules, CA, USA).

## 4. Conclusions

The prevalence of antibiotic-resistant *K. pneumoniae* with KPC genes is rapidly growing worldwide and may represent a global threat to healthcare systems in the near future. This study investigated the resistant potential of *K. pneumoniae* isolated from Makkah Province, Saudi Arabia, during the Islamic pilgrimage ‘Umrah’ and identified carbapenem-resistant genes and virulence factors. The significant findings of this study demonstrated that the isolated *K. pneumoniae* were resistant to most carbapenem antibiotics (MEM and IMI), in addition to FOX, CPM, AZT, CAZ, CTC, CIP, and AMP. Although the isolates lacked the presence of the *bla_KPC_* gene, the data indicated the presence of multiple genes encoding carbapenem factors, with the *bla_VIM_* and *bla_OXA-48_* being the most prevalent genes. The study also found many plasmids and identified numerous virulence factors in the isolated *K. pneumoniae*, which could be attributed to their resistant phenotype. The reported data in this study shed light on the nature of *K. pneumoniae,* commonly found in the religious gathering seasons in Saudi Arabia. They may serve as a reference to clinicians and scientists in the region.

## Figures and Tables

**Table 1:** MIC values of different antibiotics against carbapenemase-producing *K. pneumoniae* isolates. Antibiotic resistance (R) values were indicated according to the Clinical and Laboratory Standards Institute (CLSI) (M100-S24) [35].

Strains	FOX R ≥ 32	CPM R ≥ 16	AZT R ≥ 4	CAZ R ≥ 16	CTC R ≥ 64	CIP R ≥ 4	MEM R ≥ 4	IMI R ≥ 4	AMP R ≥ 32
	MIC (µg/mL)	MIC (µg/mL)	MIC (µg/mL)	MIC (µg/mL)	MIC (µg/mL)	MIC (µg/mL)	MIC (µg/mL)	MIC (µg/mL)	MIC (µg/mL)
K1	>1024	>1024	>1024	512	>1024	>1024	32	4	>1024
K2	>1024	512	512	>1024	>1024	>1024	32	32	>1024
K3	512	256	512	128	>1024	>1024	4	64	>1024
K4	>1024	1024	1024	>1024	>1024	>1024	128	256	1024
K5	16	<0.5	<0.5	<0.5	>1024	2	<0.5	>1024	>1024
K6	>1024	1024	>1024	>1024	>1024	>1024	128	128	>1024
K7	>1024	>1024	>1024	>1024	>1024	>1024	256	128	>1024
K8	>1024	512	1024	>1024	>1024	>1024	128	64	>1024
K9	512	512	>1024	>1024	>1024	>1024	8	2	>1024
K10	>1024	>1024	>1024	>1024	>1024	>1024	512	128	>1024
K11	>1024	512	1024	512	>1024	>1024	8	16	>1024
K12	>1024	>1024	1024	>1024	>1024	>1024	128	256	>1024
K13	>1024	1024	1024	>1024	>1024	>1024	64	256	>1024
K14	>1024	1024	>1024	>1024	>1024	>1024	256	>1024	>1024
K15	>1024	1024	>1024	>1024	>1024	>1024	512	>1024	>1024
K16	>1024	1024	>1024	>1024	>1024	>1024	256	>1024	>1024
K17	>1024	512	>1024	512	>1024	>1024	64	>1024	>1024
K18	>1024	>1024	>1024	>1024	>1024	>1024	32	256	>1024
K19	>1024	512	>1024	1024	>1024	>1024	64	512	>1024
K20	>1024	512	>1024	512	>1024	>1024	4	32	>1024
K21	>1024	1024	>1024	1024	>1024	>1024	1	8	>1024
K22	>1024	128	128	512	1024	>1024	128	>1024	>1024
K23	>1024	>1024	>1024	>1024	>1024	>1024	32	32	>1024
BAA 1705	512	64	1024	128	128	>1024	32	8	>1024

MIC: minimum inhibitory concentration, FOX: Cefoxitin, CPM: Chlorpheniramine, AZT: Azidothymidine, CAZ: Ceftazidime, CTC: Chlortetracycline, CIP: Ciprofloxacin, MEM: Meropenem, IMI: Imipenem, and AMP: Ampicillin.

**Table 2:** The frequency of carbapenemase-resistant *K. pneumoniae* isolates forming biofilm (*n* = 23).

Biofilm Pattern	Frequency % (No of Isolates/23)
Strong	0% (0/23)
Moderate	13.04% (3/23)
Weak	48.83% (11/23)
No Biofilm	39.13% (9/23)

**Table 3:** This table shows the type of samples and the patient demographics for the carbapenems-resistant *K. pneumoniae*.

Sample No.	Source of Specimen	Sample No.	Source of Specimen
1	Blood	13	Sputum
2	Wound	14	Sputum
3	Urine	15	Sputum
4	Blood	16	Sputum
5	Sputum	17	Central venous puncture
6	Urine	18	Urine
7	Wound	19	Sputum
8	Wound	20	Wound
9	Urine	21	Sputum
10	Sputum	22	Wound
11	Sputum	23	Sputum
12	Blood		

**Table 4:** Distribution of carbapenem genes in 23 isolates.

Gene	Positivity Rate (*n*/23) *n* = Number of Isolates	Positivity Rate (%)
*bla_KPC_*	0/23	0%
*bla_OXA-48_*	20/23	87%
*bla_VIM_*	23/23	100%
*bla_NDM1_*	7/23	30%
*bla_IMP_*	0/23	0%
*bla_OXA-23_*	1/23	4%

**Table 5:** Detection of carbapenemase genes in 23 isolates.

Sample	*bla_KPC_*	*bla_OXA-48_*	*bla_VIM_*	*bla_NDM_*	*bla_IMP_*	*bla_OXA-23_*	Total
K1		+	+				2
K2		+	+	+			3
K3		+	+				2
K4			+	+			2
K5			+				1
K6		+	+	+			3
K7		+	+				2
K8		+	+	+			3
K9		+	+				2
K10		+	+	+			3
K11		+	+				2
K12		+	+	+			3
K13		+	+				2
K14		+	+				2
K15		+	+				2
K16		+	+				2
K17		+	+				2
K18		+	+				2
K19		+	+				2
K20		+	+				2
K21			+				1
K22		+	+	+		+	4
K23		+	+				2

**Table 6:** Criteria of classification for biofilm formation.

Standard OD Value	Biofilm Formation
OD > 4 × ODc	Strong
2 × ODc < OD ≤ 4 × ODc	Moderate
ODc < OD ≤ 2 × ODc	Weak
OD ≤ ODc	Non

The results are presented as mean ± SD for three independent replicates. OD, optical density; ODc, optical density cut-off.

**Table 7:** PCRs Mix and amplicon sizes (bp) for detecting plasmids in *K. pneumoniae* isolates.

PCR Mix	M1	M2	M3	M4	M5	M6	M7	M8
Target Site Amplicon Length (bp)	HI1 (534)	M (741)	FIB (683)	L (854)	T (750)	U (843)	FIB KN (631)	HIB-M (570)
	HI2 (298–308)	N (514)	FIA (462)	X3 (284)	A/C (418)	X1 (370)	X2 (376)	FIB-M (440)
	HIα (159)	I2 (316)	P1 (345)	I1Y (161)	FIIS (259–260)	R (248)	FIB KQ (258)	FII (288–292)
		BO (159)	W (242)		N2 (177)	FIIK (142–148)	K (190)	X4 (172)

**Table 8:** Primer sequence and amplicon sizes (bp) for detecting carbapenem genes in *K. pneumoniae* isolates.

Gene	Primer Sequence	Amplicon Size (bp)	Reference
*KPC*	F: 5′-CGTCTAGTTCTGCTGTCTTG-3′ R: 5′-CTTGTCATCCTTGTTAGGCG-3′	798	[57]
*NDM-1*	F: 5′-GGTTTGGCGATCTGGTTTTC-3 R: 5′-CGGAATGGCTCATCACGATC-3′	621	[57]
*OXA-48*	F: 5′-GCGTGGTTAAGGATGAACAC-3′ R: 5′-CATCAAGTTCAACCCAACCG-3′	438	[57]
*OXA-23*	F: 5′-GATCGGATTGGAGAACCAGA-3′ R: 5′-ATTTCTGACCGCATTTCCAT-3′	501	[58]
*IMP*	F: 5′-GGAATAGAGTGGCTTAAYTCTC-3′ R: 5′-GGTTTAAYAAAACAACCACC-3′	232	[57]
*VIM*	F: 5′-GATGGTGTTTGGTCGCATA-3′ R: 5′-CGAATGCGCAGCACCAG-3′	390	[57]

*KPC*, Klebsiella pneumoniae carbapenemase; *NDM*, New Delhi metallo-β-lactamase; *OXA*, oxacillinase; *IMP*, imipenemase; *VIM*, Verona integron-encoded metallo-β-lactamase.

**Table 9:** Primer sequence and amplicon sizes (bp) for detecting virulence genes in *K. pneumoniae* isolates.

Gene	Primer Sequence	Amplicon Size (bp)	Reference
*Kfu*	F: GAAGTGACGCTGTTTCTGGC R: TTTCGTGTGGCCAGTGACTC	797	[60]
*WAb G*	F: ACCATCGGCCATTTGATAGA R: CGGACTGGCAGATCCATATC	683	[61]
*Uge*	F: TCTTCACGCCTTCCTTCACT R: GATCATCCGGTCTCCCTGTA	534	[61]
*rmpA*	F: ACTGGGCTACCTCTGCTTCA R: CTTGCATGAGCCATCTTTCA	516	[61,62]
*Fim H*	F: TGCTGCTGGGCTGGTCGATG R: GGGAGGGTGACGGTGACATC	688	[63]
*mag A*	F: GGTGCTCTTTACATCATTGC R: GCAATGGCCATTTGCGTTAG	1282	[61]
*CPS*	F: TATTCATCAGAAGCACGAGCTGGGAGAAGCC R: GTCGGTAGCTGTTAAGCCAGGGGCGGTAGCG	418	[64]

*Kfu*: *Klebsiella* ferric iron uptake, *Uge*: UDP galacturonate 4-epimerase, *rmpA*: regular mucoid phenotype, *FimH*: fimbrial adhesin, *mag*: mucoviscosity-associated gene, and *Cps*: capsular polysaccharide.

## Data Availability

The data presented in this study are available within the article.

## Supplementary Materials

The following supporting information can be downloaded at: https://www.mdpi.com/article/10.3390/antibiotics11111627/s1. Table S1: The raw data and biofilm formation level of all 23 *K. pneumoniae* isolates, along with the ATCC reference strain (700603); Table S2: Detailed results of all replicons appeared in all 23 *K. pneumoniae* isolates.
