# Supplementary material for: Identification and Characterization of Plasmids and Genes from Carbapenemase-Producing Klebsiella pneumoniae in Makkah Province, Saudi Arabia
Source: Antibiotics (Basel). 2022 Nov 15;11(11):1627. doi: 10.3390/antibiotics11111627 (PMC9686665; doi:10.3390/antibiotics11111627)
Supplement: Supplementary file 1 [file antibiotics-11-01627-s001.zip › antibiotics-2006026-supplementary.pdf]

Supplementary Materials

S1. Biofilm Formation of *K. pneumoniae*

Table S1. The raw data and biofilm formation level of all 23 *K. pneumoniae* isolates, along with the ATCC reference strain (700603).

Isolate No.	Average	SD	Biofilm Level	Isolate No.	Average	SD	Biofilm Level
**1**	0.097	0.006	Weak	**14**	0.130	0.003	Moderate
**2**	0.129	0.062	Moderate	**15**	0.120	0.003	Weak
**3**	0.056	0.001	No Biofilm	**16**	0.058	0.003	No Biofilm
**4**	0.072	0.003	Weak	**17**	0.057	0.001	No Biofilm
**5**	0.096	0.021	Weak	**18**	0.056	0.002	No Biofilm
**6**	0.114	0.005	Weak	**19**	0.056	0.001	No Biofilm
**7**	0.106	0.004	Weak	**20**	0.057	0.001	No Biofilm
**8**	0.096	0.020	Weak	**21**	0.058	0.001	No Biofilm
**9**	0.055	0.002	No Biofilm	**22**	0.077	0.007	Weak
**10**	0.136	0.009	Moderate	**23**	0.054	0.001	No Biofilm
**11**	0.071	0.021	Weak	**ATCC 700603**	0.153	0.004	Moderate
**12**	0.099	0.012	Weak	**Negative**	0.057	0.002	No Biofilm
**13**	0.119	0.031	Weak				

S2. Detection of Plasmids by PCR-Based Replicon Typing (PBRT)

Table S2. Detailed results of all replicons appeared in all 23 *K. pneumoniae* isolates.

Sample	Replicons	Total of plasmid
**K1**	*I1Y, FIIK, FIB KQ, FII*	4
**K2**	*I1Y, FIIK, FIB KQ, IncA/C L, HIB-M*	5
**K3**	*I1Y, FIB-M, FIB KN, R, HIB-M*	5
**K4**	*I1Y, FIIK, FIB KQ, , HIB-M*	5
**K5**		0
**K6**	*FIIK*	1
**K7**		0
**K8**	*FIIK*	1
**K9**	*A/C*	1
**K10**	*FIIK*	1
**K11**		0
**K12**	*FIB KQ*	1
**K13**	*FIIK, FII*	0
**K14**	*FII, FIIK*	2
**K15**	*I1Y, R, FIB KQ, A/C, FIIS, FII*	6
**K16**	*I1Y, N2, IncW L, A/C, FIIS, U, FII*	7
**K17**	*I1Y, X1, IncW L, A/C, FIIS, FIB KN, FIB-M, HIB-M*	8
**K18**	*I1Y, X1, IncW L, A/C, FIIS, FIB KN, FIB-M, R, HI3, HIB-M*	10
**K19**	*I1Y, X1, IncW L, A/C, FIIS, FIB KN, FIB-M, FIIK, HIB-M*	9
**K20**	*I1Y, X1, IncW L, A/C, FIIS, FIB KN, FIB-M, HIB-M*	8
**K21**	*I1Y, X1, IncW L, A/C, FIIS, FIB KN, FIB-M, HIB-M*	8

**K22**	*I1Y, N2, IncW L, A/C, FIIS, FIIK, FII, FIB KQ*	8
**K23**	*I1Y, FIIK, FIB KN, FIB-M, HIB-M*	5
